# Advocate cultivation of academic ethics: why is it necessary?

**DOI:** 10.12688/f1000research.20640.2

**Published:** 2020-02-19

**Authors:** Sok-Ja Janket, Jukka Meurman, Eleftherios P. Diamandis

**Affiliations:** 1 Section of Translational Oral Medicine, Forsyth Institute, Cambridge, MA, 02142, USA; 2 Department of Oral and Maxillofacial Diseases, University of Helsinki, Helsinki, Finland; 3 Pathology and Laboratory Medicine, Mount Sinai Hospital, Toronto, Toronto, Ontario, M5T 3L9, Canada

**Keywords:** Academic, Ethics, Behaviour, Education, Faculty

## Abstract

We teach and practice ethical behavior with all clinical and research activities. Notably, we are well educated to treat the subjects participating in research studies with high ethical standards. However, the ethics of interacting with colleagues, or with junior faculty members, are neither well defined nor taught. Dealing with junior faculty has parallels to dealing with vulnerable research subjects such as children, mentally or physically challenged groups, prison inmates or army recruits. Like any other vulnerable population, lower-ranking faculty members are often at the mercy of department chairs or other higher-ranked faculty members. Herein we present some potentially unethical or unfair examples related to academic research. Our goal is to educate the academic community of conceptual paths and to prevent similar untoward occurrences from happening in the future. Unethical behaviors related to sexual misconduct have already been described elsewhere and are not included in this manuscript.

## Introduction

In 2014, the World Medical Association celebrated the 50 ^th^ anniversary of the Declaration of Helsinki ^1, 2^. This Declaration forms the basis for all later ethical proclamation relating to how “we care for the persons who are our patients, for those who seek advice, and protect those who volunteer in research studies” ^2^. Through repeated training in human research subject protection, researchers recognize the malevolence of exposing unsuspecting prison inmates to syphilis bacteria to study immune responses or use organs from executed criminals for transplantation without their consent. However, the boundaries of ethics in collaboration with colleagues or with the junior faculty members are not well-defined nor discussed. We, hence, discuss the importance of teaching and following a code of ethics in interactions between colleagues in the academic setting. 

One of the pioneers of ethical education, Dr. Bertolami, wisely pointed out that our ethics curricula in biomedical education are informative, but fail to be formative and transformative ^3^. The reason is that ethics courses teach mainly abstruse concepts that are vague and intangible. Thus, as he asserts, they stay informative at best but are not strong enough to change a person’s views. Indeed, “ethics” is often used interchangeably with “morality” ^4^, but ethics has more to do with a “philosophical concept” and morality has more to do with human actions ^5^. Aristotle used the word “ *hexis*” to describe moral virtue in relation to ethics ^6^. Because *hexis* is an active condition, “virtue”, in this context, implies an action. Again, we arrive at the conclusion that ethics must bring action, namely being transformative ^7^. Therefore, it is necessary to teach ethics in a more action-based and example-based format so that learners can apply ethical judgment in their everyday activities.

**Figure 1.**
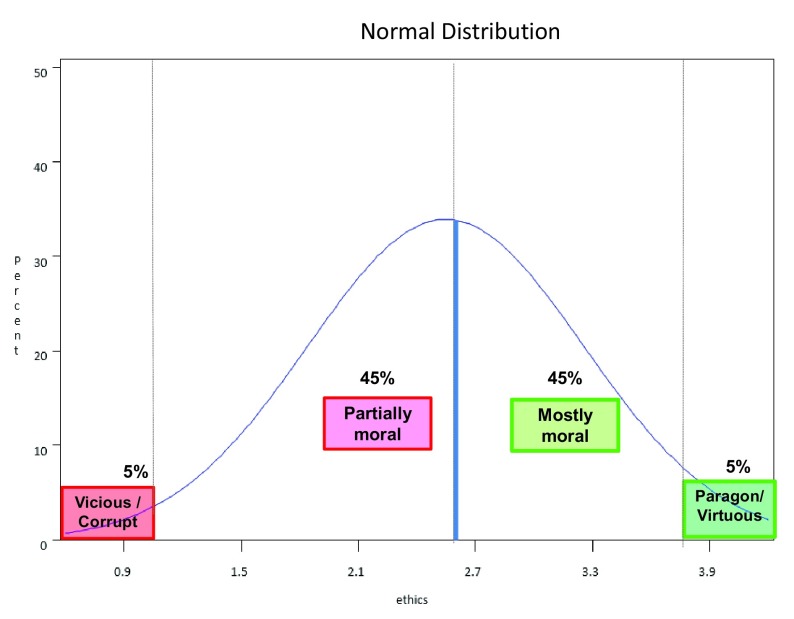
The ethical bell curve. This graph was modified with permission from Bertolami ^7^.

**Figure 2.**
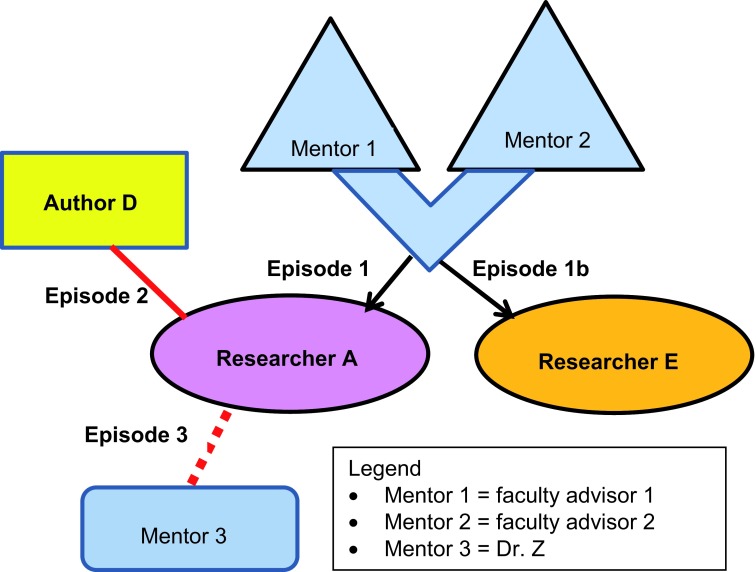
Episodes and associated persons flow chart.

 Dr. Bertolami also presented the Aristotelian grouping of an ethical bell curve ^7^. We created a similar bell curve using our data and labeled them according to Aristotelian/Bertolamian categorization ( Figure 1). We surmise that very few individuals in health science belong to the ‘vicious’ group, where serial killers or white-collar embezzlers may be included, or the ‘paragon’ group where Mother Theresa-like persons will belong. We arbitrarily consider them outliers belonging to the upper and lower 5% and also speculate that most researchers belong in the middle 90% ^7^. Thus, the ethical transgressions we observe in biomedical research fall into the broad area of the middle 90% and it is not easy to determine whether the observed conduct is truly unethical, marginally unethical or not unethical. Clearly, there is a need to define the boundaries between ethical and unethical conduct in academia and give actual examples that underscore such demarcation. We suggest that special committees be formed for each academic institute whose responsibilities are to oversee any deviations from the acceptable ethical boundaries and make recommendations to remediate any alleged ethical transgressions. Let us explore some examples derived from our own experiences and consider solutions to resolve or prevent similar incidences. The relationships of the persons involved in the presumed ethical transgressions are illustrated in Figure 2.

We have summarized our experience in academic ethics and suggested remedies to prevent future unethical transgressions in Table 1.

**Table 1.** Summary of potential ethical issues and study points in biomedical research.

Ethical issues	Realities	Our perspectives
I. Vertical relationship between senior faculties and lower ranking mentees make the mentees vulnerable for their career development if they express different views from their advisers scientifically or personally.	a) Mentors exercise their power beyond scientific realms to retaliate against mentees in the review processes of grants and manuscripts. b) Consciously or subconsciously, “The Sins of expertness” are prevalent ^8^. c) Mentee’s next employment can also be negatively impacted by the mentors.	1. We need to set the boundaries of ethical conduct when mentors and mentees have different opinions on the same issue. 2. We need to define and consider strategies to prevent the potential abusive conduct of senior faculty members. 3. The “behind the door” consults of the job seekers’ former employer or advisors should be discouraged. It should be open and transparent to the job seekers.
II. Recognizing and giving credit to previously published intellectual property (IP) even if it involves mundane and universal concepts.	a) If the IP involves well-known, ordinary facts, it is easy not to give credit to the authors who previously published the construct. b) Plagiarism must include “fail to acknowledge the authors who published the said IP contents earlier”.	1. Recognize plagiarism is not limited to word-for-word copying. 2. Respect and quote other authors who published similar concepts no matter how obvious the concept may be. 3. Ethical behavior is not instinctive. It must be learned.
III. In collaboration of Senior and junior faculty members in grant writing, consider the lower ranking faculty members are a part of vulnerable populations.	a) Senior faculty request lower ranking members for basic knowledge to build the rationale while assuming the role of PI. b) Collaboration is not doing the PI s work.	1. The role of PI (principal investigator) is acquiring abundant knowledge on the subject and building the study rationale. Merely reading prior similar studies without critically evaluating the methodology will bound to repeat the short-comings of the previous studies. 2. Co-investigators should not perform the activities described above as the role of PI. Co-investigators can provide specific knowledge for the study, i.e., flow- cytometry or PCR procedures. 3. In the ideal collaborative environment, PI will set up rationale and design the detailed action plan, while co-investigators will provide specific expertise.
IV. Ethical issues in grant review: • Often reviewers do not have sufficient knowledge related to the topic. • Many proposals deal with one small aspect of total science and often not related to causality.	a) Reviewers are usually funded researchers and are more or less obligated to review. b) Recognize the potential conflicts of interest when a proposal is contrary to the reviewers’ own research theorem. c) Potentials do exist that ‘Peter funds Paul’s grant this time and Paul reciprocates next time’.	1. Make conscious efforts to evaluate the science only. 2. Recognize the limitation of one’s knowledge and conduct accordingly. 3. If we consider the benefits to the society, causality should be the most important criterion in a grant review.

## Episode 1a: “Challenging your senior faculty could be hazardous to your career!”

Dr. Sackett humorously described ^8^ how senior faculty members can wield undue power over lower-ranking members of academia beyond the scientific context ^8^. This is more palpable when the advisees reveal the flaws of senior members’ research methodology ^8^. One example of such concealed retaliation can be found in the case of Researcher A.

In late 1996, several small dental studies published a significant association between periodontitis and cardiovascular diseases (CVD) ^9, 10^. Admittedly, these are clinical studies fraught with varying degrees of deficiencies and small sample sizes. Interestingly, two large studies from a famous institution (θ University) were published with null results (no association). Thereafter, the whole world was eager to believe the null results because of the institution’s fame, seemingly excellent methodology and the large sample sizes (~55,000 and ~22,800 study subjects).

While learning meta-analysis methodology as a Master of Public Health student at the same famous θ University, Researcher A chose this topic as a class project to evaluate the evidence without *praejudicium* (prejudged or pre-decided without being fairly heard). Researcher A truly believed that making a clear objective conclusion would benefit the research community and his faculty advisors would be pleased with such an endeavor. Researcher A did the project so well and received a standing ovation from fellow classmates and high praise from the instructor. The results were “pure science” without any influence of personal feelings or sense of affiliation. The conclusion was that there was a small but significant association between periodontitis and CVD. More importantly, the mathematical analyses have proven that seemingly poorly conducted studies overestimated the risk by only 13%, while ostensibly well-conducted studies underestimated the risk by 29.7%, thus appearing to be more biased than smaller studies. Later, Researcher A informed his faculty advisor of the results of meta-analysis. This faculty adviser (FA 1) was one of the lead authors of the null-result reporting papers from the θ University. FA 1 showed Researcher A the doctoral thesis which reported similar 30% attenuation in the risk estimates some 4 years earlier. Thus, what Researcher A observed in the meta-analysis was in agreement with the faculty adviser’s thesis. We note that the said faculty adviser did not realize the deficiencies of own thesis which reported 30% underestimation of the true risk. Rather, this faculty adviser thought that the culpability resided in other studies. Consequently, this faculty member wrote several opinion papers criticizing the other small studies.

Since the publication of this meta-analysis, which became a sentinel study cited over 560 times, Researcher A’s manuscripts and grant proposals have been harshly criticized and rejected numerous times presumably by these faculty advisors. Because of their institutional fame, these faculty advisors benefited from the privilege of being the most sought-after reviewers. After being rejected so many times, Researcher A’s grant was enigmatically funded when these faculty advisors were facing a legal action from an anti-compound λ group.

## Episode 1b: How to reconcile when student’s research collides with the advisers’ theory?

Researcher E was a classmate of Researcher A at the same θ university and both were mentored by the same faculty advisors. Researcher E collected data for over 5 years and analyzed them. Researcher E found that her results suggested that exposure to compound λ increased the risk of osteosarcoma in adolescent boys. When Researcher E was about to publish the results, said advisors strongly recommended not publishing them citing that her data were of poor quality. This obstruction of the doctoral thesis by said faculty advisors leaked to an advocacy group opposed to the use compound λ and public uproar followed. Moreover, θ University, fearful of any future litigation from the anti-compound-λ group, forced the senior of the two faculty advisors (FA 2) to retire. Collaterally, the poor data collection method as revealed by meta-analysis prevented the younger of the two faculty advisers from winning tenure. Although neither Researcher A nor Researcher E had anything to do with the anti-compound-λ group’s protest on these faculty advisers, the animosity from the faculty advisers towards Researchers A and E deepened without due cause. Although the results showing the association of compound λ and the risk of osteosarcoma were finally published, Researcher E could not find a research position after 6 years of intensive PhD training. Perhaps, the behind-the-scenes evaluation might have had something to do with this researcher’s thwarted research career.

Here, it is worth discussing the objectivity of covert consultation of mentors without the knowledge of mentee’s especially when mentees have differing scientific views from that of their mentors. It is common practice that prospective employers, including post-doctoral research positions, contact the former employer or former faculty advisors about the prospective employee. The sad part is that this important process takes place without the knowledge of the researchers seeking positions. Thus, the nascent researchers have no idea why their job applications were declined. Consequently, what Voltaire had said some 300 years ago was proven true in 2000s: “ *It is dangerous to be right in matters on which the established authorities are wrong.*” 

Our perspectives:

1. To strengthen the fidelity of science, differing opinions should be encouraged. This includes divulging the flaws of faculty members’ methodologies.
2. We find the action of a prospective employer contacting faculty advisor without informing the position seeking researchers unethical although it is quite prevalent. If such an action is elected, they should contact the mentor in the presence of the mentee. Researcher A did not know why his numerous job applications were rejected until one prospective employer called the FA1 in the presence of Researcher A. We find this covert evaluation beyond the written letter of recommendation is despicable and should be abolished.
3. We recognize that it is only human to harbor ill-feelings towards the mentees who exposed one’s own research flaws. We, therefore, suggest that mentors should totally extricate themselves from giving any evaluations of those mentees who provoke ill-feelings. In the case of these two faculty advisors, the younger FA1 delegated reviews of the mentees to the senior FA2 who is more prone to be harsher due to their powerful positions. Technically, FA1 relinquished review of mentees but handed it to a crueler evaluator.

Also, take note that until the publication of the said meta-analysis, no one understood the cause for the conflicting results in the periodontitis-CVD association although all the researchers involved were top-notch scientists. This becomes another topic of unethical behavior by another researcher in *Episode 2*.

## Episode 2: Do two bad ethical transgressions cancel each other out?

As stated above, no one who read the studies reporting the association between periodontitis and CVD knew the cause for the conflicting reports. When Researcher A had proven that non-differential misclassification was the cause of the null results using meta-regression, many people had an “aha!” moment. An “aha!” moment occurs when a simple principle that many people already know explains a complex phenomenon. For this reason, it is easy to claim another person’s intellectual property (IP) that brought an “aha!” moment as their own.

In essence, Researcher A proved that the two large studies with meticulous confounding adjustment were more biased than the small, seemingly poorly conducted studies. Because it is expensive to conduct clinical examinations when a sample size is in the tens of thousands, the larger studies used a questionnaire to diagnose periodontitis. When a less-than-precise method, such as a questionnaire, is used, it causes non-differential misclassification. Non-differential misclassification is an elementary epidemiologic concept that can be explained in simple terms as follows: The truly diseased are included in the non-diseased group and some non-diseased persons are included in the diseased group because the measuring instrument was not precise. The result is reduced contrast which, in epidemiology, is referred to as “attenuation of the risk estimate”. It is a well-known epidemiologic principle that non-differential misclassification moves the risk estimates towards the null. However, connecting the dots of non-differential misclassification to the results of the “periodontitis-CVD relationship” was Researcher A’s IP. Let us peruse what has transpired after Researcher A’s meta-analysis.

Researcher A wrote in the meta-analysis in 2003: “…potential for underestimation by 2 large epidemiologic studies because of the non-differential misclassification stemming from the use of questionnaires instead of clinical examination of periodontal disease.”

Author D wrote quoting Researcher A (not for non-differential misclassification but for heterogeneity) in 2005: “potential misclassification of periodontitis in studies not employing periodontal probing for assessment of periodontitis……. Indeed, the attenuation of relative risk estimates due to such misclassification can be quite dramatic”.

In 2008, Author D wrote quoting his own 2005 publication: *“*potential misclassification of periodontitis in studies not employing periodontal probing for assessment of periodontitis…Indeed, the attenuation of relative risk estimates due to such misclassification can be quite dramatic”. **

Careful readers will recognize that Researcher A in 2003, and author D in 2005, wrote the same scientific construct with small changes in wording. Is Author D not obligated to quote Researcher A for this sentence because Researcher A has already published the very concept 2 years prior? Clearly, Author D had read Researcher A’s article when he quoted Researcher A for “heterogeneity” which is a far less important piece of information than misclassification. It appears that Author D used a two-step “bait and switch” tactic to divert the contest from Researcher A by quoting for something peripheral to the issue and made the more important concept of “non-differential misclassification” in the periodontitis-CVD relationship as his own IP.

Researcher A read the above phrase which Author D wrote in 2008 and thought “Whoa, this is exactly what I wrote in 2003. Did he quote me”? But, it was found that Author D quoted his own 2005 paper. So Researcher A traced back to his 2005 publication and discovered that Researcher B quoted Researcher A for very nonessential “heterogeneity” but not for the important “non-differential misclassification”. A majority of meta-analyses report heterogeneity. Thus, it appeared that the construct of “non-differential misclassification attenuated the risk estimate in the periodontitis-CVD association” became his own IP in a two-step ‘bait and switch’ tactic. First, neutralize any protest from Researcher A by quoting the article for an unimportant fact and leaving the real important construct out. And in the second step, he quoted his own paper reporting the important construct without citing the Researcher A, who published this concept some years earlier. This two- step unethical misappropriation of someone else’s IP could be due to unintentional neglects. This is why we need ethical training to recognize that a subtle transgression may be a form of plagiarism.

Our perspectives:

1. We must educate ourselves to give credit to prior studies. Even if the concept involves a basic and mundane principle, the first person who used this mundane construct to solve the scientific queries should be given credit.
2. We need to recognize that ethics is a human construct that needs to be taught.
3. Ethical learning curve is applicable to everyone. We first thought citing Aristotle’s Nicomachean Ethics would be adequate citation for our categorization of human ethics ( Figure 1). However, after further thinking, we determined citing Bertolami would be appropriate because he used the modern terminologies which made us easy to understand Aristotle’s categorization.
4. We advocate that plagiarism should include not only “word for word” copying but also using other people’s concepts and ideas without appropriate citation.

Let us consider another example of a study using a mundane fact to explain a weighty conclusion. In 2012, a study conducted by a group of physicians and epidemiologists reported in a leading medical journal that dental x-rays might increase brain cancer meningioma in children ^11^. Later, a group of investigators reported that the exposure assessment of the original study ^11^ was done by self-report and thus the results are not trustworthy ^12^. Almost everyone with elementary knowledge in epidemiology knows that self-reported data are notoriously unreliable. Do we see the parallels of flawed methodology in the usage of questionnaire and self-report? They are both extremely imprecise, causing biased results. Anyone who has basic epidemiologic knowledge and read the original article ^11^ would have recognized the flawed exposure assessment. Nevertheless, it is our opinion that the first researcher(s) who identified and published this flaw in the original study ^11^ should be given credit as the owner of that IP ^12^.

## Episode 3: Watch your foes carefully and watch your friends even more carefully!

Researcher C has been regarded as an expert on the relationship between oral infections and CVD and had published over 30 papers on the topic. Researcher C’s mentor, Dr. Z, known for her fairness and thoughtfulness helped Researcher C’s career transition from clinician to researcher. In appreciation of this mentor’s guidance, Researcher C included the mentor in a number of oral health and CVD-related publications. Subsequently, Dr. Z decided to write a grant proposal for assessing the relationship between oral health and CVD, assuming the role of the principal investigator (PI). In essence, the mentor became Researcher C’s competitor. The mentor appears to think that including researcher C as a co-investigator is fair compensation. However, Researcher C felt that Researcher C should be one of the co-PIs. The differing views on fair compensation caused a rift in their once close relationship.

Sadly, ideas, and the associated IP can easily be purloined without any credit given to the source during the discussion because copyright or patent is not possible for these ideas. The concepts Researcher C shared with the mentor during a conversation became the mentor’s idea without any credit given to Researcher C. The concept that forms IP is the fundamental starting point for any scientific endeavor and should come from the PI, not the co-investigators. Often, in open discussions, Dr. Z requested members to provide opinions on how to approach the methodology. This is particularly troublesome when senior faculty members urge junior faculty members to donate their IP although the senior faculty members are the principal investigators. If junior faculty members decline, the senior faculty members are in the position of negatively affecting the junior faculty members’ careers. In this context, the junior faculty members are no different than the vulnerable research study subjects. Therefore, every effort should be made to protect junior faculty members in research collaborations.

Our perspectives:

1. An unspoken rule in research is that mentors should stay clear of the mentee’s research domain. This is because mentors are in a position of power and if they compete against mentees, they are in more advantageous locus. A good mentor will place mentees as PIs and put themselves as co-investigators.
2. If the senior faculty is the PI, their role is to develop a study rationale and the research strategy. This requires much reading and critically evaluating prior studies not only supporting his/her study objectives but opposing said objective. The role of PI (i.e., building a study rationale and planning the execution strategy of the rationale) should never be delegated.

## Episode 4: Ethics in grant proposal writing

Currently, few researchers consider ethics when writing grant proposals. However, this should change, because a poorly conceived study will waste limited research resources and reduces the probabilities of other worthy studies getting funded. Thus, all researchers have a collective responsibility to conceptualize, prepare and execute a study plan that will benefit society.

Most researchers are under tremendous pressure from the institution with which they belong and “getting funded” becomes the ultimate impetus for writing grants without adequate learning and preparation. What was once, “publish or perish”, nowadays becomes “funded or fade away”. In these conditions, the foremost role of the principal investigators (PIs) is accumulating adequate knowledge and developing a rationale so that the co-investigators will follow the well-planned study protocol. Too often, PIs are senior faculty members and sometimes have minimal knowledge of the study’s objectives. Using the position of authority, they delegate important tasks that clearly belong to the role of PI to junior faculty members who do not have enough experience or knowledge. The consequence of this dereliction of duty is an ill-prepared grant that might waste resources.

The vertical relationship between senior and junior faculty members in collaboration harbors potential for an abuse of authority. The delegation of tasks can be perceived as an order. Often, senior researchers use words to appeal to the guilty conscience of junior faculty members, such as “this work is good for all of us and good for the institution”. Basically, this can be interpreted as “work hard without getting the credit you deserve”. The unspoken message is “if you do not do what I ask you to do, you are acting against all of us and against the organization we serve.” Forced donations, as suggested by senior faculty members, whether monetary or IP, should be discouraged. We decry sweat shops in South East Asia for their abusive labor practice but very few are speaking against intellectual sweat shops in academia. Is it because the perpetrators and victims both belong to the highly educated echelons of society? Abusive labor practice is abusive, no matter where it occurs.

## Episode 5: Ethical issues in grant review

There are additional problems with the grant review process. The reviewers are usually those who were funded in the similar domain of research and they often have a hidden agenda to fund the application in the same direction as their own. For example, if the reviewer received funding to investigate oral cancer in relation to human papilloma virus (HPV), it is highly unlikely that this reviewer will fund an application aiming to prove that HPV was not a cause of oral cancer. Thus, whatever biases the reviewer has, will be perpetuated and any proposal that is not concurrent with reviewer’s research direction will likely not be funded. These biases are often unconscious, and the reviewer may not realize that she/he may be blocking a new thesis. Although the National Institute of Health (NIH) is looking for “innovative” research, our experience tells us that reviewers rarely support any innovative idea that may take away their own funding potentials. Others have expressed similar concerns on these non-financial conflicts of interest ^13^. This is a clear conflict of interest but the grant review system has been in operation as far as we can remember. Thus, we do not know whether the application has no scientific merit or is opposite of the reviewer’s research theory.

The qualifications of reviewers can also sometimes be problematic. If a study involves human-level outcomes, it will be reviewed by epidemiologists who usually do not conduct molecular-level research. And yet, their review determines the fate of many well-thought-out translational research projects. Often, the value of the scientific merit is secondary to the submitter’s personality. Someone who never spoke against another scientist’s flaws will get funded with mediocre science, while scientists who tried to expand our scientific understanding by pointing out deficiencies in other’s research may not be so lucky.

Wasteful funding may be attributed to the funding agencies’ demand for “innovation” as a key element in grant proposals. “Innovative” research innately encourages charting untested waters. At present, buzzwords ending with “-ome”, i.e., genome, microbiome, proteome, transcriptome, metabolome, etc. are in vogue. These studies are looking at the whole collective constructs, be it microorganisms (over 10 trillion in a human body), genes (25,000+ human genes) or proteins (10,000 to potentially billions in a human body). First, it is nearly impossible to pinpoint any one or several of these as causative of a human pathology. Moreover, the investigators use an easy to access compartment such as the fecal microbiome to estimate the causative microbes in the gut, which may not be similar ^14^. This is a major scientific misstep because feces are the end results of intestinal activities, not the cause for them ^15, 16^. Consequently, many millions of research dollars have been spent but we are not certain of the benefits these results may bring to society. Reviewers will not support a brand new idea citing “the lack of preliminary data” even in pilot grants. Without a pilot funding, the idea will not be tested at all. The ideas with proven track record will be shunned as “not innovative enough.” Once again, Voltaire was correct in saying: “ *When the idea is new, people say it is not true. When finally it is proven to be true, people say it is not new.*” 

Our perspectives:

1. The key ethical consideration in grant writing should be whether the grant proposal will improve human health. Thus, selecting causal relationship that will engender better health for all humans is the crucial.
2. The most important tenets of ethical grant review is reviewing the potential for human health improvement. Too often, many studies that will never reach human level results guzzle up a major portion of research funds.
3. There is no easy way to protect the rights of junior faculty members when they collaborate with senior investigators. Educating scientific community on this issue and the senior members of faculty may voluntarily protect the junior faculty members.

## Concluding remarks

We must all take pride in not surrendering to the overwhelming power of the established dogma. As the late columnist Charles Krauthammer said “ *If you are going to leave the medical profession because you have something to say, you betray your whole life if you don’t [say] and if you don’t say it honestly and bluntly.*” For all those who spoke out to make academic research just and fair, whether their honesty and bluntness will contribute to the betterment of academic research ethics will not be known immediately. Only history will tell.

## Data availability

No data are associated with this study.
