# Supplementary material for: Advocate cultivation of academic ethics: why is it necessary?
Source: F1000Res. 2020 Feb 19;8:1830. Originally published 2019 Oct 30. [Version 2] doi: 10.12688/f1000research.20640.2 (PMC7043133; doi:10.12688/f1000research.20640.2)
Supplement: Peer review history

# Peer review: Reviewer response for version 2

**DOI:** 10.5256/f1000research.24404.r60248

**Published:** 2020-02-19

**Authors:** Konstantinos Drosatos

**Affiliations:** 1 Center for Translational Medicine, Department of Pharmacology, Lewis Katz School of Medicine, Temple University, Philadelphia, PA, USA

I concur to index the revised version of the article in its present form.

I confirm that I have read this submission and believe that I have an appropriate level of expertise to confirm that it is of an acceptable scientific standard.

# Peer review: Reviewer response for version 1

**DOI:** 10.5256/f1000research.22701.r56447

**Published:** 2019-12-20

**Authors:** Julie Shaw

**Affiliations:** 1 The Ottawa Hospital, Ottawa, ON, Canada

Here, the authors describe several ethical considerations in academics. This is an interesting topic and the ethical issues related to academic are seldom acknowledged. This is largely an opinion piece, with the conclusions those based on the authors' opinions.  Unconscious bias in academics is an important topic that should be addressed.

 My only comment for consideration to address is for the authors to somehow make clearer the relationships they describe in their "episodes". I found it difficult to keep track of the various relationships. Perhaps there is a way to do the pictorially.

I confirm that I have read this submission and believe that I have an appropriate level of expertise to confirm that it is of an acceptable scientific standard.

# Peer review: Reviewer response for version 1

**DOI:** 10.5256/f1000research.22701.r56143

**Published:** 2019-11-25

**Authors:** Konstantinos Drosatos

**Affiliations:** 1 Center for Translational Medicine, Department of Pharmacology, Lewis Katz School of Medicine, Temple University, Philadelphia, PA, USA

In this article, Sok-Ja Janket et al. present several hypothetical cases that resemble real-life situations and pertain to ethical challenges that researchers deal with on frequent basis primarily at the mentor-mentee level. The authors are experienced researchers with significant training record. Thus, both their experience and academic caliber render them suitable to offer unbiased and objective point of view on such an important matter.

 Here are my suggestions that may improve the content of the article:

1. The use of single letters as hypothetical names is somewhat confusing, especially because multiple exams are listed in the text. Instead of using Researcher Dr. A, it would be easier to process designations such as Dr. Mentor, Dr. Postdoc, Ms. Student etc.
2. Although it is understandable that not all paradigms can be included in the article, it would be important to add one more scenario on "Ethics in assigning leadership positions to junior faculty" e.g. The Society of Research is creating an Early Career Committee to address aspects that pertain to junior faculty. The leadership of the Society of Research has to nominate junior members to operate the ECC. Dr. Past Trainee, who had pursued his post-doctoral training with the President of the Society of Research is selected instead of Dr. Award, who received last year's Outstanding Junior Faculty Award... [CONTINUE]
3. The article would have been even more meaningful if the authors devoted a section on certain and clearly described suggestions about institutional action that needs to be taken. The section should go beyond description of principles and include even some examples of action that has been taken by leadership to serve ethical standards and the authors may be aware of. 
4. I am not positive of the suggestion that research proposals should be reviewed in the light of potential for improvement of human health. Various research findings, such as siRNA that was discovered in plants ^1^ were translated to health-related applications long time after their discovery, which might not even imply translatability.  

MINOR: p.6; Typo in Episode 2: Replace "had proved" with "had proven"

I confirm that I have read this submission and believe that I have an appropriate level of expertise to confirm that it is of an acceptable scientific standard, however I have significant reservations, as outlined above.

## References

[1] : A species of small antisense RNA in posttranscriptional gene silencing in plants. *Science*.1999;286(5441) : 10.1126/science.286.5441.950 950-2 10.1126/science.286.5441.950 10542148
